# A multiligament, internal brace, coaptationless stifle reconstruction technique for feline stifle luxation

**DOI:** 10.1111/vsu.70092

**Published:** 2026-02-27

**Authors:** Sebastian C. Knell, Philipp A. Schmierer, Antonio Pozzi

**Affiliations:** ^1^ Clinic for Small Animal Surgery, Vetsuisse Faculty University of Zurich Zürich Switzerland; ^2^ Tierklinik Posthausen Posthausen Germany

## Abstract

**Objective:**

To describe a novel, coaptationless surgical technique for the treatment of multiligament stifle injuries (MLSI).

**Study design:**

Retrospective case series.

**Animals:**

A total of 23 cats presenting with a rupture of two or more ligaments stabilizing the stifle.

**Methods:**

Specific surgical techniques were used to reconstruct each deficient ligament. The TightRope procedure was utilized to treat the cranial cruciate ligament and the lateral collateral ligament injuries. Intra‐articular reconstruction was adopted for the caudal cruciate ligament. The medial collateral ligament was reconstructed using knotless anchors. Postoperative joint immobilization was not employed in any case. Clinical evaluations were performed up to 6 months postoperatively including the feline musculoskeletal pain index (FMPI).

**Results:**

Initial stabilization of MLSI using the described techniques was achieved in all cases. Major complications occurred in six cases: four involved recurrent caudal cruciate ligament instability, and two involved medial patellar luxation. All complications were resolved with revision surgery, except in one case where arthrodesis was required. At follow‐up evaluations, lameness ranged from grade 1 to undetectable. FMPI scores ranged from 0.95 to 1.0.

**Conclusion:**

The surgical technique described offers an effective approach for managing MLSI in cats without the need for temporary immobilization, provided that all injured ligaments are appropriately reconstructed. Specifically, reconstruction of the caudal cruciate ligament should be performed when deficient, as it may help mitigate the risk of postoperative complications.

**Clinical significance:**

This coaptationless technique allows for effective restoration of stifle stability in feline patients, with low complication rates and good clinical outcomes.

## 1 INTRODUCTION

Stifle luxation or multiligament stifle injury (MLSI) is a severe traumatic injury associated with ligament ruptures leading to severe instability.^1^, ^2^, ^3^, ^4^ The high degree of energy involved in these injuries leads to rupture of two or more ligaments.^5^ Avulsion fractures, meniscal injuries and capsular tears are common.^1^, ^2^, ^3^, ^4^, ^5^ Surgical stabilization is recommended, including repair or reconstruction of the ligaments using prosthetic material,^1^, ^2^, ^3^, ^4^, ^6^, ^7^ combined with postoperative joint immobilization using coaptation, transarticular external skeletal fixators, or transarticular pinning.^1^, ^2^, ^3^, ^4^

MLSIs are associated with substantial morbidity due to persistent instability, progressive osteoarthritis (OA) and reduced range of motion. Reported overall complication rates exceed 60%, with major complications occurring in more than 30% of cases.^2^, ^3^, ^4^ While the use of postoperative immobilization has been recommended to protect the prosthetic and repaired ligaments, it has also been associated with impaired ligament healing in experimental models, and complications including implant failure, bone fracture, loss of joint mobility and OA.^2^, ^3^, ^4^, ^8^

Braided ultra‐high‐molecular‐weight polyethylene suture and knotless bone anchors allow earlier mobilization and improved outcomes in people with multiligament knee injuries.^9^ The biomechanical advantages of employing knotless constructs with bony anchorage and high‐strength braided suture material for stifle stabilization in dogs and cats include reduced creep, increased construct stiffness, and greater resistance to elongation when compared with conventional techniques that rely on soft‐tissue anchorage and knotted sutures.^12^, ^13^ In dogs and cats, surgical techniques utilizing bony anchorage and knotless techniques as described in people for different indications, offer superior strength, which can allow a shortening of the postoperative joint immobilization and achieve better clinical results.^12^, ^13^, ^14^, ^15^

This report describes the outcome and complications of an anatomical reconstruction technique for MLSI without postoperative joint immobilization.

## 2 MATERIALS AND METHODS

### 2.1 Study population

The medical records database was searched for cats with multiligament stifle injury treated with a new anatomical reconstruction at the veterinary teaching hospital University of Zurich and the Tierklinik Masans between 2019 and 2024. Cats were included in the study if the documentation of the initial clinical examination and surgical report and the pre‐ and postoperative radiographs were available, and a follow‐up evaluation of at least 6 months was performed. Cats that did not return for a follow‐up evaluation, including radiography, at least 6 months after surgery were excluded.

Multiligament stifle injury was defined as an injury to at least two major stabilizing ligaments (cruciate or collateral ligaments).^1^, ^2^, ^3^, ^4^, ^5^ Other injuries such meniscal or capsular tears were listed. Cats were grouped according to the injured ligaments.

MLSIs were classified based on the type of injuries: (I) Cranial cruciate ligament (CrCrL) and collateral ligament (CL) injuries. (II) Cranial and caudal (CdCrL) cruciate ligament injury. (III) Cranial and caudal cruciate ligament and collateral ligament injuries.

### 2.2 Medical records and radiographic review

Medical records were reviewed to collect information on breed, sex, bodyweight, number of days from trauma, and age at time of surgery. Information was recorded regarding the injured limb, the presence of any other injury and all surgical procedures and implants involved. Pre‐, postoperative and follow up orthogonal radiographs were reviewed by a board‐certified surgeon (SCK).

### 2.3 Surgical technique

**FIGURE 1:**
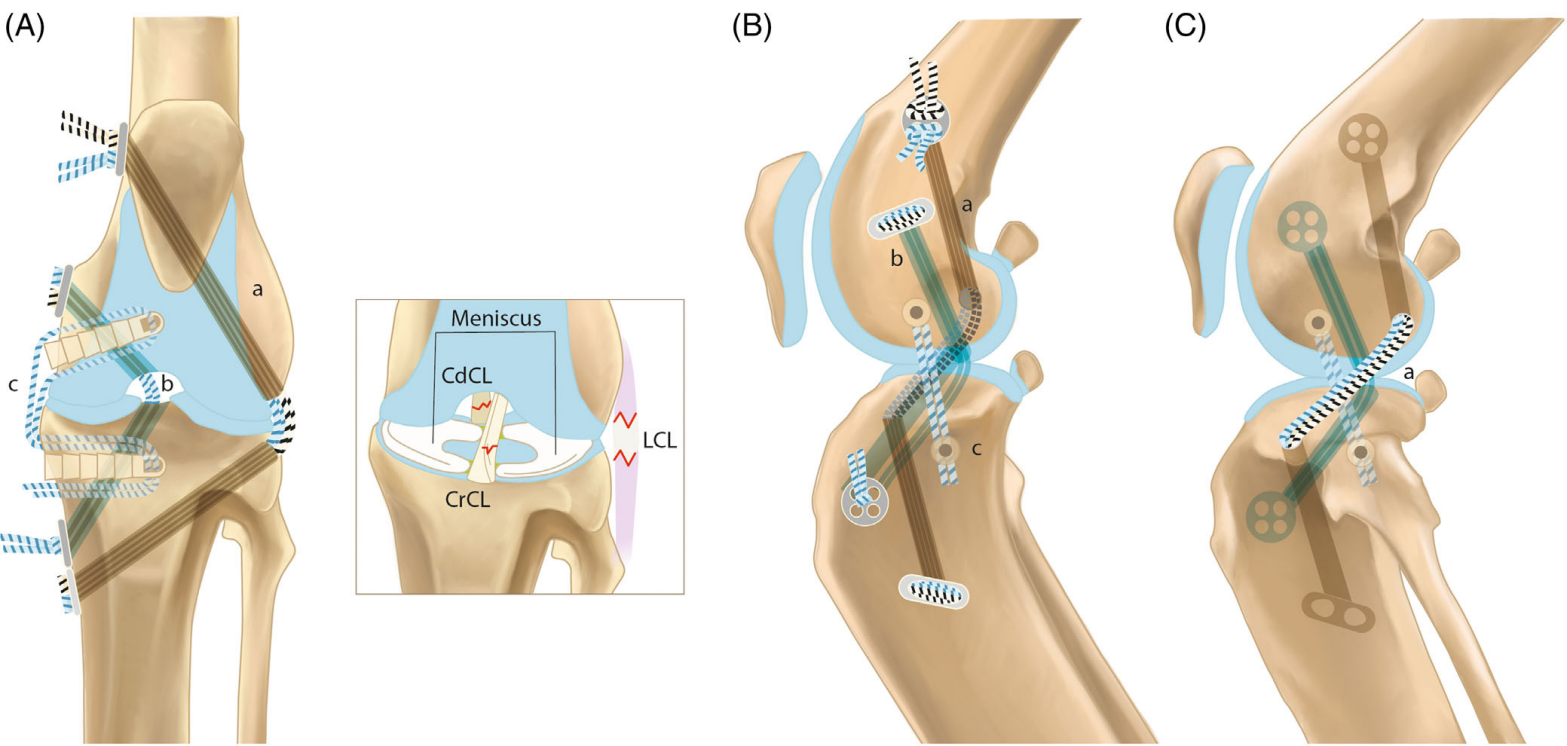
Schematic drawing of the multiligament anatomical reconstruction for multiligament stifle injury (MLSI) in cats. (A) Cranio‐caudal view of the stifle. The intra‐articular reconstruction of the caudal cruciate ligament (CdCrL) is shown (b) alongside the extra‐articular sutures (a) stabilizing the cranial cruciate ligament (CrCrL) and the lateral collateral ligament (LCL) deficiencies. On the medial side (c), the medial collateral ligament (MCL) reconstruction is depicted using knotless anchors. A close‐up view highlights the menisci, both cruciate ligaments, and the LCL. For clarity, these structures are omitted in the subsequent drawings to improve the visibility of the reconstructions. (B) Medial view of the stifle. This view illustrates the reconstructions of the MCL (c), CdCrL (b), and CrCrL (a). The suture material running through the bone tunnel is shown as a shadow for clarity. (C) Lateral view of the stifle. The lateral view highlights the course of the extra‐articular CrCrL reconstruction (a), which also serves to stabilize the LCL insufficiency.

**FIGURE 2:**
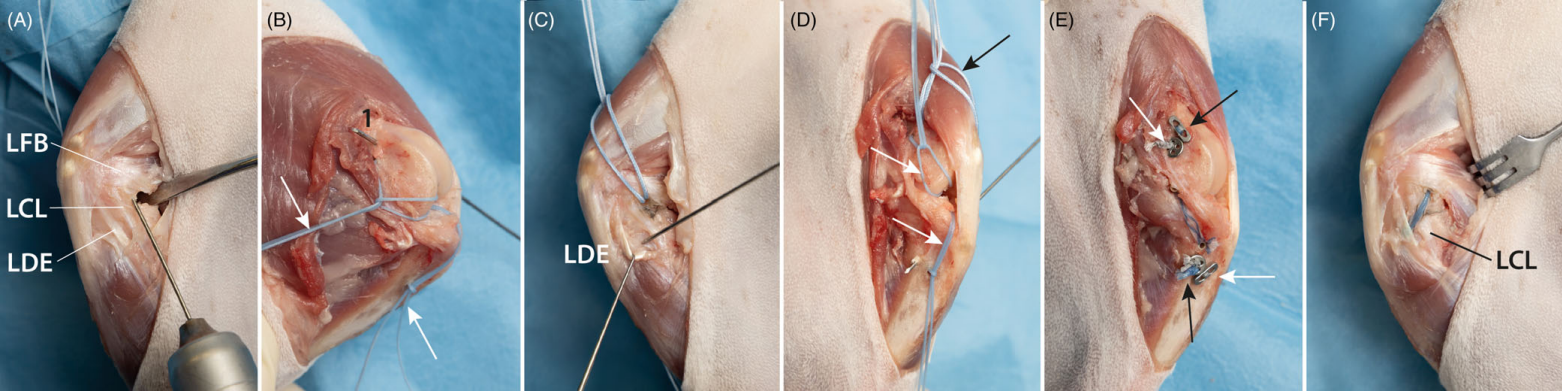
Extra‐articular reconstruction of the cranial cruciate ligament (CrCrL). (A) Lateral view of the stifle joint. A periosteal elevator is used to retract the lateral fabella (LFB). A K‐wire is inserted at the caudal edge of the lateral femoral condyle, positioned caudal to the lateral collateral ligament (CL) and distal to the fabella, to create the proximal bone tunnel for the extra‐articular CrCrL reconstruction. The k‐wire is aimed proximal to the patella exiting on the medial femoral side. (B) The previously placed sutures for CdCrL reconstruction are visible (white arrows). The exit point (1) of the femoral tunnel for the CrCrL should lie proximal to the CdCrL tunnel and at least 10 mm distal to the patella to avoid interference. The K‐wire is then overdrilled using a 2.0 mm cannulated drill bit. (C) The long digital extensor (LDE) is retracted with a small hook. The K‐wire marks the entry point of the tibial tunnel, just caudal to the extensor groove, and is directed toward the caudal edge of the medial surface of the tibia. Once correct positioning is confirmed, the K‐wire is overdrilled using the same cannulated drill bit. (D) Medial view of the surgical field. The previously placed CdCrL sutures (white arrows) and the femoral tunnel of the CrCrL (black arrow) are visible. The tibial tunnel for the CrCrL exits caudal to that of the CdCrL. (E) Medial view showing the tightened sutures: The intra‐articular CdCrL reconstruction (white arrow) and the extra‐articular CrCL reconstruction (black arrow). (F) Lateral view of the completed CrCL reconstruction. Due to its anatomical course, the CrCrL graft can also provide stabilization in cases of lateral collateral ligament (LCL) deficiency.

**FIGURE 3:**
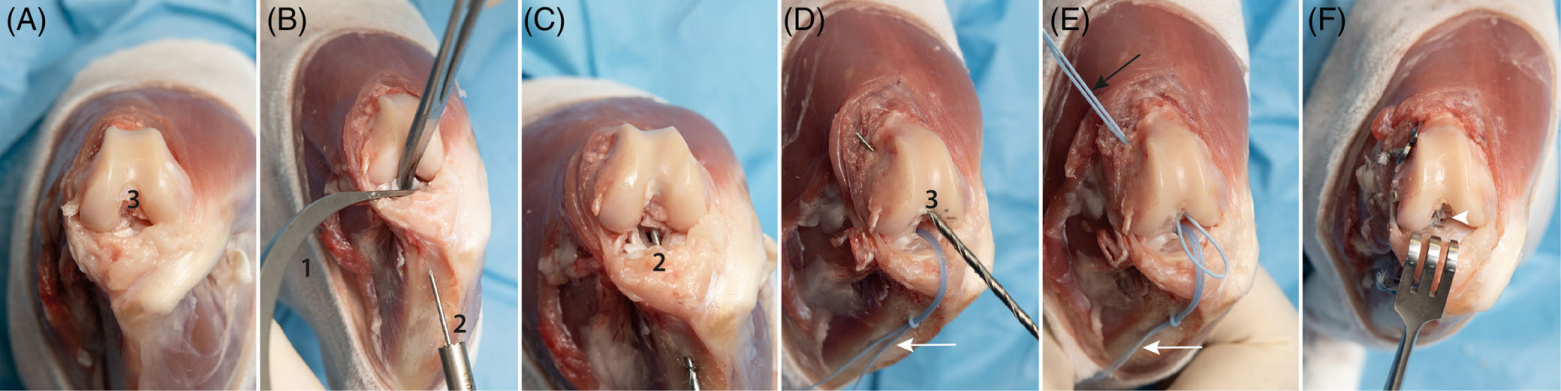
Intraarticular reconstruction of the caudal cruciate ligament (CdCrL). (A) A medial parapatellar approach to the stifle joint is shown. The CdCrL has been transected at its femoral origin (3). (B) A hemostat has been placed caudal to the tibial plateau to subluxate the tibia cranially, allowing improved access to the CdCrL's tibial insertion. An aiming device (1) is positioned at the tibial insertion site, with a K‐wire (2) directed toward the medial surface of the tibia, where the tunnel is started. To prevent slippage due to the steep angle of insertion, it is recommended to predrill the cis cortex before advancing the K‐wire. The optimal insertion point is mid‐tibia, approximately 1–2 cm distal to the joint line. (C) The K‐wire has been advanced through the tibia, exiting at the CdCrL's insertion (3). A 2.0 mm cannulated drill bit is then used to overdrill the tunnel. (D) A temporary suture loop (white arrow) has been passed through the drilled tibial tunnel. Using the same cannulated drill bit, a second tunnel is created by overdrilling a K‐wire placed at the CdCrL's femoral origin (black arrow), exiting on the medial side of the femur. A second suture is shuttled through this femoral tunnel to facilitate suture passage and identification of the two tunnels. (E) Two temporary suture loops have been inserted. One is passed through the femoral tunnel (black arrow) and one through the tibia tunnel (white arrow). (F) An anteroposterior view of the reconstructed multiligament stifle injury (MLSI) shows the intra‐articular portion of the caudal cruciate ligament (CdCL) graft (white arrowhead).

**FIGURE 4:**
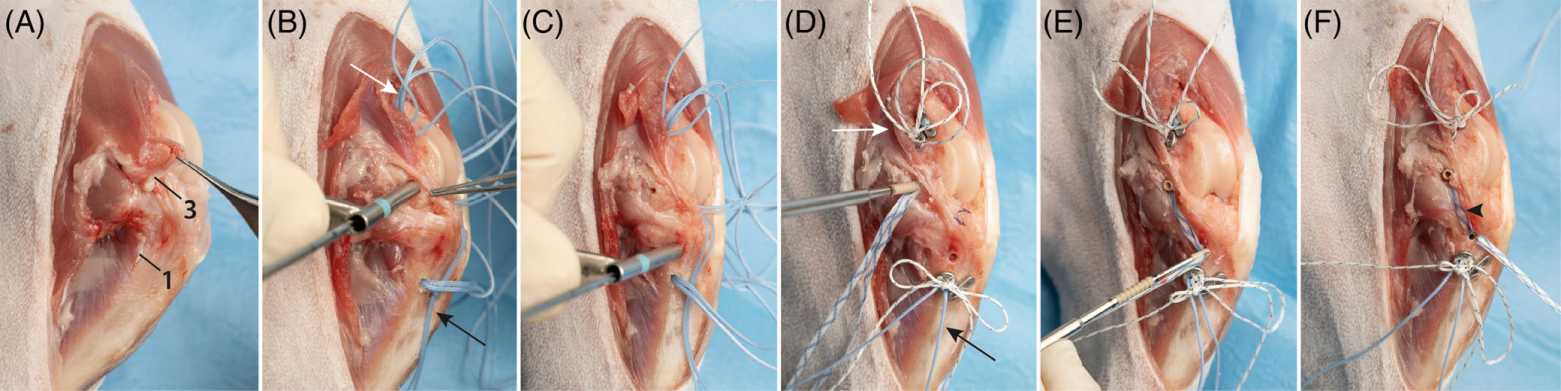
Knotless reconstruction of the medial collateral ligament (CL). (A) Medial view of the stifle showing the ruptured medial CL. The anatomical origin (3) and insertion (1) of the medial CL are indicated. (B) A bone tunnel is created at the medial CL origin using a 2.5 mm drill bit. The diameter of the drill hole should correspond to the size of the interference anchor (2.4 mm in this case) and the suture material (1.2 mm suture tape). The drill should be angled toward the proximal pole of the patella to ensure adequate bone stock and avoid impingement on the intercondylar notch. In the image, the drill is initially placed perpendicular to the bone to prevent slippage and then angled appropriately. Preplaced sutures from the proximal cranial cruciate ligament (CrCrL) (white arrow) and caudal cruciate ligament (CdCrL) (black arrow) are visible. (C) The medial CL insertion site at the medial tibia is drilled. Care must be taken to avoid interference with the tunnels of the CrCrL and CdCrL. This must also be considered when placing the corresponding tibial tunnels of the CdCrL and CrCrL. (D) A 2.4 mm interference anchor is placed into the bone tunnel. It is critical to pre‐tighten the CrCL and caudal cruciate ligament (CdCL) to ensure correct joint alignment and to prevent malpositioning of the medial CL in a non‐neutral position. Preplaced sutures from the proximal CrCrL (white arrow) and CdCrL (black arrow) are visible and pretightened. (E) The proximal anchor is inserted after shuttling the suture tape through its eyelet. The anchor is then advanced into the bone tunnel. (F) Final view of the completed medial CL reconstruction using knotless interference bone anchors (black arrowhead), demonstrating anatomical restoration without the need for external knots.

**FIGURE 5:**
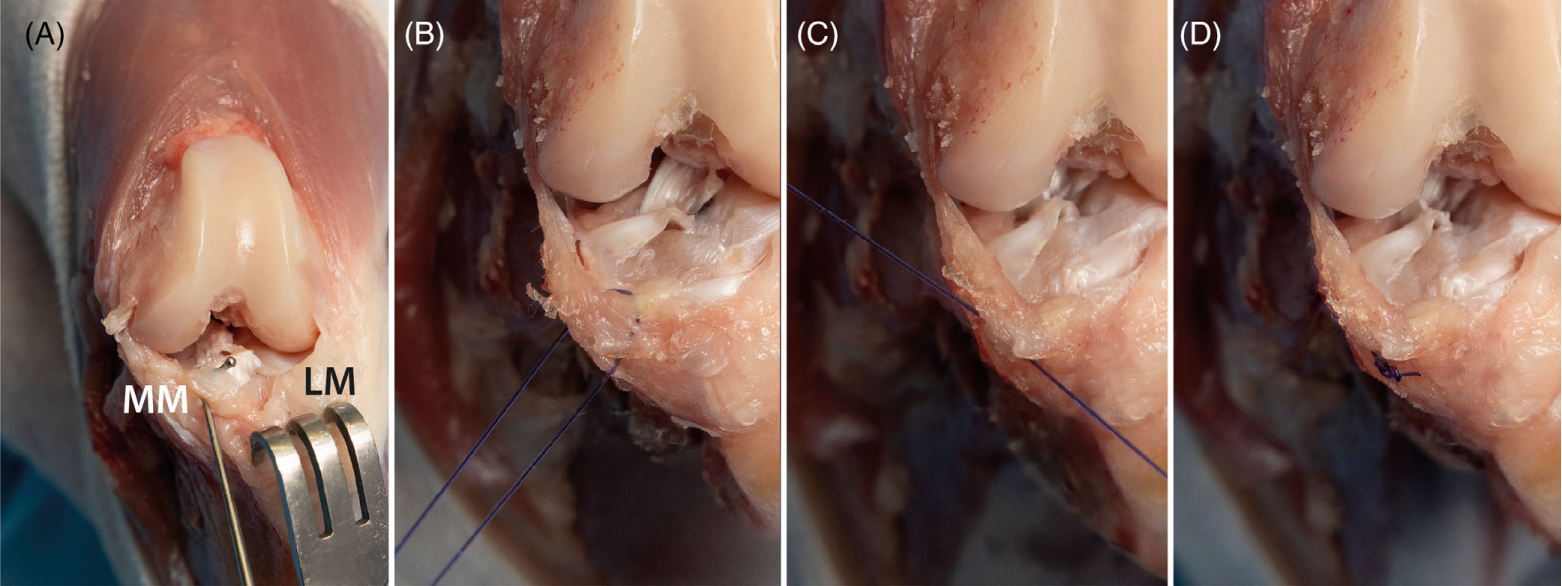
Meniscal repair. (A) Craniocaudal view of the stifle joint displaying the medial (MM) and lateral (LM) menisci. The medial meniscus is torn and retracted using a palpation hook for better visualization. A Senn retractor is applied to advance the tibia cranially and improve access. (B) The torn meniscus is reapproximated using monofilament suture material. (C) The suture is securely tied, restoring the meniscus to its anatomical position. (D) Final view of the repaired medial meniscus in its physiological alignment.

The goal of the surgical technique was to achieve a reconstruction of every ligament as anatomically as possible and to repair the collateral ligaments when possible (Figure 1):

- An extra‐articular reconstruction of the CrCrL with bone tunnels and multifilament sutures (MiniTightRope, Arthrex GmbH, Munich, Germany) to neutralize both cranio‐caudal and rotational instability, which is common in acute CrCrL injuries (TightRope (TR) Procedure). The technique was also used to stabilize laxity associated with lateral CL rupture (Figure 2).
- An intra‐articular reconstruction of the CdCrL with bone tunnels to achieve anatomical alignment of the stifle and a balanced and functional stifle with the CrCrL reconstruction. The CdCrL rupture was treated conservatively in the first six cases. After recognizing caudal tibial subluxation in two cases the surgical technique was modified, and the intra‐articular reconstruction was performed in all cases with CdCrL rupture in conjunction with the CrCrL reconstruction (Figure 3).
- An anatomical reconstruction of the medial CL with a knotless anchor (PushLock 2.5 or 2.9, Arthrex GmbH) to optimize multiplanar stability (Figure 4).
- Meniscal repair using an inside‐out suture technique to preserve joint function.^15^ Non‐repairable meniscal tears were treated with meniscectomy (Figure 5).

The detailed surgical steps are described in Appendix A and in the images illustrating the technique.

### 2.4 Anesthesia and analgesia

Cats were premedicated via IV or IM injection, typically including dexmedetomidine (2–5 μg/kg, Dexdomitor, Provet, Lyssach, Switzerland), methadone (Methadon Streuli^©^, Streuli Pharma, Uznach, Switzerland), ketamine (1 mg/kg Ketasol, Graeub, Bern, Switzerland) and propofol (Propovet, Zoetis, Delemont, Switzerland) to effect to induce anesthesia. Anesthesia was maintained with isoflurane, oxygen, and fentanyl infusion (5–10 μg/kg/h Sintenyl; Sintetica, Mendrisio, Switzerland). All cats received perioperative antibiotics (Cefazolin 22 mg/kg IV, Sandoz, Rotkreuz, Schweiz), which were continued no longer than 24 h post‐surgery unless other injuries required antibiotic treatment.

Postoperative analgesia consisted of buprenorphine (0.02 mg/kg every 6 h, Bupaq P, Streuli, Uznach, Switzerland) for 3–5 days after surgery and nonsteroidal anti‐inflammatory drugs (Metacam, Boehringer, Basel, Switzerland) for 5–7 days after surgery.

Postoperative mild to moderate swelling of the operated limb was addressed with cold packing every 4 h for 20 min for the first 48 h.

### 2.5 Owner instructions

Owners were instructed to confine the cats in a limited area without furniture for 8 weeks. No postoperative coaptation was used in any of the cases. Additionally, owners were instructed by a certified physiotherapist on how to perform passive range‐of‐motion exercises.

### 2.6 Clinical and radiographic assessment

#### 2.6.1 Pre‐ and postoperative and follow‐up assessment

All cats underwent physical and complete orthopedic examination preoperatively and 24 h postoperatively. Follow‐up examination at 6–8 weeks and 6 months included physical and orthopedic examination with subjective assessment of gait (grade 0–5; grade 0 = no lameness, grade 5 = severe lameness) and range of motion (ROM) evaluated in comparison to the sound limb (normal to slightly decreased, moderately decreased, or severely decreased). One of the authors, a board‐certified surgeon, assessed each cat.

#### 2.6.2 Radiographic assessment

Radiographs were performed upon admission, immediately postoperatively, and 8 weeks and 6 months postoperatively to evaluate the bone tunnels (widening or change in tunnel appearance compared to postoperative) and OA progression. Postoperatively, implant, tunnel position and joint reduction were assessed and classified as appropriate or unsatisfactory. Joint reduction was evaluated by comparison with a normal feline stifle radiograph.

On follow‐up radiographs, implant position and integrity were compared with immediate postoperative images to identify any changes. Furthermore, osteoarthritis (OA) was assessed subjectively and graded as mild, moderate, or severe. All radiographic evaluations were performed by a board‐certified (SCP) surgeon.

#### 2.6.3 Feline musculoskeletal pain index

The functional outcome and quality of life at home was evaluated using the North Carolina State University's College of Veterinary Medicine, feline musculoskeletal pain index (FMPI). Owners were asked to complete the FMPI^16^ at each re‐check examination, ensuring that the same person assessed the cat on each occasion. Owners were not aware of their previous scores at the re‐check FMPI.

#### 2.6.4 Complications

The intra‐ and postoperative complications, and any surgical revisions were recorded. Complications were categorized as minor (not requiring additional surgical or medical treatment to resolve), major (complication or associated adverse effects requiring further surgical (1) or medical (2) treatment), and catastrophic (complication or associated adverse effects that caused permanent unacceptable impairment of function, was directly related to death, or prompted euthanasia).^17^, ^18^

#### 2.6.5 Outcomes

The lameness score and ROM obtained with the orthopedic examination, the type and rate of complications, the progression of radiographic OA and the FMPI results were the four criteria used in this study to report the functional outcome.^18^ In addition, based on the type of complication, owner questionnaire responses, and surgeon evaluations, stifle joints were categorized as having an overall successful or unsuccessful outcome. A successful outcome was defined when no catastrophic complications occurred, and the cat was described by the owner as having full or acceptable postoperative return to function based on FMPI ≤1, no pain at stifle palpation and a lameness score ≤1/5.^5^ An unsuccessful outcome was defined as any stifle joint not meeting the criteria for a successful outcome.^18^ Stifle joints that underwent revision surgery that subsequently failed or had to be amputated or arthrodesed were defined as having an unsuccessful outcome.

### 2.7 Statistical analysis

Descriptive statistics were used to report the demographic and outcome data.

The Shapiro–Wilk test was used to assess normal distribution of the data (age, weight, FMPI, and sex). According to normality assessment, mean ± SD or median and interquartile range (IQR) were reported.

Commercially available software was used to analyze the data (Graphpad Prism, Graphpad, Boston).

## 3 RESULTS

### 3.1 Study population

Medical records from 23 cats (11 spayed females, 12 neutered males) met the inclusion criteria. The mean age was 5 years (range: 2–11 years), and the mean bodyweight was 5.1 kg (range: 4.0–6.5 kg). The cats presented 2 ± 1.5 days after injury. A total of 23 stifles with MLSI were evaluated (10 right, 13 left), with lesion distribution summarized in Table 1.

**TABLE 1:** Summary of outcomes and complications of the cases with MLSI.

Case No.	Weight	Age	Side	Classification group	Meniscal injury and (treatment)	Complication and type of revision	Lameness score 6 months post Op	ROM 6 months	FMPI 6 months
1	4.5	2	R	III	AMM (resected)		0	Normal	0.95
2	4	3	R	I	AMM (resected)		0	Normal	0.91
3	6.5	2	L	I	BHT MM (resected)		0	Slightly reduced	0.91
4	4.8	5	L	I	AMM (repaired)		0	Normal	0.8
5	4.2	5	L	I	BHT MM (resected)		0	Normal	0.8
6	5.8	4	R	I	AMM (resected)		0	Slightly reduced	0.95
7	4.4	11	L	III	AMM (repaired)		0	Normal	0.95
8	5.2	3	R	I	BHT MM (resected)	MPL corrected by imbrication	0	Normal	0.91
9	4.5	4	L	I	AMM (repaired)		1	Normal	0.91
10	5.5	8	R	III	BHT MM (resected)	CdCL intraarticular repair	0	Normal	0.95
11	4.8	5	R	I	AMM (repaired)		0	Normal	0.98
12	5.2	6	R	I	AMM (repaired)		1	Normal	0.95
13	5.5	2	R	III	AMM (repaired)	CdCL intraarticular repair	0	Slightly reduced	0.98
14	4.8	11	R	II	BHT MM (resected)		0	Slightly reduced	0.95
15	5.4	10	L	II	AMM and ALM (resected)		0	Normal	0.98
16	6.7	8	L	III	AMM (resected)		1	Normal	0.95
17	6.0	10	R	III	AMM (repaired)	CdCL intra‐articular repair	1	Normal	0.95
18	5.6	5	R	I	BHT MM (resected)		0	Normal	0.96
19	5.2	7	L	I	AMM (resected)	MPL corrected by imbrication of joint capsule	0	Normal	1.00
20	4.2	4	L	III	AMM (repaired)		0	Normal	0.98
21	5.0	4	R	III	BHT MM (resected)		0	Normal	0.98
22	6.1	6	R	I	AMM (repaired)		0	Normal	0.98
23	5.2	6	L	III	AMM (repaired)	Caudal subluxation diagnosed 4 weeks postoperatively, revised by arthrodesis	4/5	No ROM	n.a.

*Note*: Group I (cranial cruciate ligament and collateral ligament injuries), group II (cranial and caudal cruciate ligament injury), group III (cranial and caudal cruciate ligament and collateral ligament injuries).

Abbreviations: AMM/ALM, avulsion medial/lateral meniscus from joint capsule; BHT, bucket handle tear; CdCL, caudal cruciate ligament; CdCrl, caudal cruciate ligament; CrCrl, cranial cruciate ligament; FMPI, feline musculoskeletal pain index; L, left; LM, lateral meniscus; MLSI, multiligament stifle injury; MM, medial meniscus; MPL, medial patellar luxation; R, right; ROM, range of motion.

Classification of injuries was as follows: Group I: *n* = 12, Group II: *n* = 2 and Group III: *n* = 9.

Intraoperatively, capsular disruption was present in all cats. Medial meniscal damage was identified in all but one case (22/23) and the lateral meniscus was involved in one case. This case had both a medial and lateral meniscal injury. An avulsion of the medial meniscus from the joint capsule was the most frequent meniscal injury (15/22). This injury was repaired in 10 cases (10/15). The decision not to perform a repair was based on surgeons' judgment and often due to subjective insufficient strength of the repair. The remaining meniscal injuries were treated by partial meniscectomy.

### 3.2 Clinical and radiographic assessment

#### 3.2.1 Pre‐ and immediate postoperative assessment

All cats initially presented with severe, non–weightbearing grade 5‐5 lameness. Orthopedic examination revealed cranio‐caudal and rotational instability in all cases. Medial instability was detected in 21 cats, and lateral instability in four cats; these findings corresponded with intraoperative observations.

Within 24 h postoperatively, all cats regained weightbearing on the affected limb. The stifles were stable and had normal ROM.

#### 3.2.2 Radiographic assessment

**FIGURE 6:**
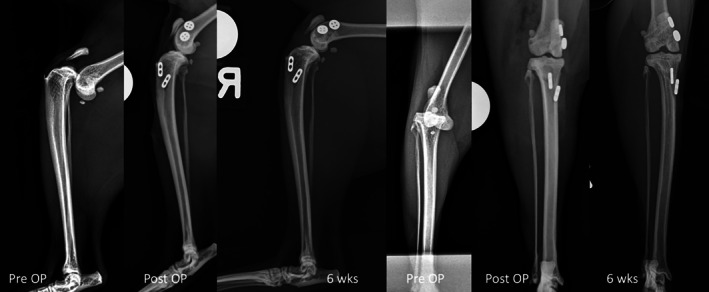
Clinical case where the CrCL, the CdCL were reconstructed including the follow up images at 8 weeks. The images show appropriate joint alignment, and correct implant position. Note the orientation of the bone tunnel of the cranial cruciate ligament (CrCrL) in relation to those of the caudal cruciate ligament (CdCrL) in both femur and tibia.

Immediate postoperative radiographs confirmed appropriate joint alignment and correct implant placement in all cats (Figure 6). Four cats presented between 1 and 2 weeks postoperatively because of lameness and radiographs showed caudal tibial subluxation. These four cats underwent surgical revision (see section on complications).

At the 8‐week follow‐up, tibial tunnel widening associated with the TR procedure reconstruction was observed in one cat, increasing from 2.5 mm immediately postoperatively to 3.7 mm. This cat showed no clinical signs of pain, instability, or lameness.

Another cat demonstrated apparent widening of the femoral anchor tunnel for medial collateral ligament reconstruction, though without clinical abnormalities. Mild OA was noted in the two cats that showed persistent mild lameness and reduced ROM; however, both retained acceptable clinical function.

At the 6‐month follow‐up, no new cases of tunnel widening were identified, and no progression of existing widening was noted. Progressive OA was documented in two cats, both of which were already affected at the 8‐week recheck.

#### 3.2.3 Feline musculoskeletal pain index (FMPI)

FMPI data were available for 21 of 23 cats at both the 8‐week and 6‐month follow‐ups. Scores indicated good to excellent function in all cases, ranging from 1 to 0.8 at 8 weeks (mean = 0.95 ± 0.04) and from 1 to 0.95 at 6 months (mean = 0.98 ± 0.03), with 1 representing the best score (Table 2). Cats requiring revision surgery also had good functional outcomes post‐revision based on FMPI. FMPI data were not available for the cat that underwent arthrodesis.

**TABLE 2:** The incidence of complications and outcomes reported for each group.

Group	Number of cats	Lameness grade (mean ± SD) at 8 weeks postoperatively (0–5)	Number of cats with reduced ROM 8 weeks postoperatively	FMPI at 8 weeks postoperatively median (IQR)	FMPI at 6 months postoperatively median (IQR)	Number (%) of cats that required revision surgery
I	12	0.33 ± 0.45	3	0.95 (0.18)	0.93 (0.2)	2 (16%)
II	2	1 ± 0	1	0.96 (0.01)	0.97 (0.03)	0
III	9	0.55 ± 1.0	3^a^	0.95 (0.07)	0.95 (0.03)	4 (44%)

*Note*: Group I (cranial cruciate ligament and collateral ligament injuries), group II (cranial and caudal cruciate ligament injury), group III (cranial and caudal cruciate ligament and collateral ligament injuries).

Abbreviations: CdCrL, caudal cruciate ligament; CL, collateral ligament; CrCrL, cranial cruciate ligament; FMPI, feline musculoskeletal pain index; IQR, interquartile range: ROM, range of motion.

^a^ The cat revised with an arthrodesis was not available.

#### 3.2.4 Complications

Minor postoperative complications occurred in 2/21 cats, consisting of mild incisional erythema with superficial skin reactions.

Major postoperative complications were observed in 6/23 stifles (26.6%). All cats required surgical intervention:

- Caudal stifle subluxation occurred in four cases. Three of these four cases had a CdCrL that was not reconstructed in the initial surgery. Three were successfully treated with intra‐articular CdCrL reconstruction, resulting in stable joints. In one case, identified 4 weeks postoperatively, stifle arthrodesis was performed.
- Medial patellar luxation (MPL) of grade 2/4 developed in two cats due to dehiscence of the joint capsule closure. Both were corrected using medial capsular release and lateral imbrication, with return to full function.

#### 3.2.5 Follow up and overall outcome

At the 8‐week follow‐up, when including also the cats that underwent revision surgery, 12 cats exhibited a mild grade 1/5 lameness, and 10 cats were clinically normal. The cat that underwent an arthrodesis had a mechanical lameness (3/5) but no pain. Seven cats demonstrated a mildly decreased ROM compared with the contralateral limb. By the 6‐month follow‐up, no cats displayed any lameness except the cat that underwent a stifle arthrodesis.

Overall, 22/23 cats met the criteria for a successful outcome, after surgical revision in three cases. The cat that underwent arthrodesis was assigned an unsuccessful outcome.

## 4 DISCUSSION

In this clinical report cats with MLSI treated with a reconstruction of each ligament had a successful outcome without postoperative immobilization. Cats with a ruptured CdCrL that was not reconstructed required a surgical revision. Managing the cats without postoperative joint immobilization allowed a return to normal ROM with minimal OA progression 6 months postoperatively. We found the technique feasible and repeatable by following precise steps for tunnel drilling and suture tightening. Familiarity with knotless anchors is crucial to perform this technique successfully.

Most cats in the study had a fast recovery and an excellent joint function based on the recheck examinations and the FMPI scores. We developed the technique following the principles of internal brace, introduced by Gordon McKay for the treatment of ankle injuries in people.^19^, ^20^ Internal brace consists of a high‐strength synthetic reconstruction to reinforce a ligament, either repaired or not, or a graft during healing. The internal brace acts as a secure belt for the healing tissue, allowing immediate stability. In our study we applied this concept to the medial and lateral CL, which were repaired or sutured to the joint capsule before applying the knotless reconstruction. Our clinical results confirm that the concept of internal brace can be adopted in animals, offering similar advantages to people such as immediate weight bearing without joint immobilization.

We developed an intra‐articular reconstruction of the CdCrL, which was found critical to counteract the forces from the CrCrL reconstruction and reestablish anatomical alignment. We interpret this finding as a proof of the principle that reconstruction of all ligaments is important to achieve optimal stability in multiple planes and directions.

In our study conservative management of the CdCrL in MLSI increased the risk of recurrent instability, caudal tibial subluxation and poor limb function. This finding is in line with the approach to knee multiligament injuries in people, where all ligaments are reconstructed in a single‐stage surgery to achieve the best functional results.^9^, ^21^

The TR procedure was successful in treating CrCrL and lateral collateral ligament injuries. The TR procedure has several advantages compared to traditional techniques, including a strong polyblend suture and a more isometric position of the anchorage points of the prosthetic suture.^13^, ^22^ These features make the TR procedure an excellent choice for MLSI as it offers superior strength compared to standard extracapsular techniques. As it spans the lateral aspect of the stifle joint close to the insertion of the lateral collateral ligament, we observed that this technique may also function as a prosthetic repair of the lateral collateral ligament supported by the original description of the technique, where the landmarks are approximately the origin and insertion of the collateral ligaments.^13^

We found that knotless bone anchors combined with strong polyblend sutures offered a new, low‐profile treatment option for the reconstruction of the medial CL. The stability of a knotless CL reconstruction was proven to be superior to knotted techniques for tarsal luxations^12^ and based on several biomechanical studies.^10^, ^11^, ^23^ However, controlling tension can be more difficult with knotless anchors than with knots. In this application, we found that the reconstruction should not be overtightened because of the risk of reducing ROM. In addition, planning carefully the exit of the bone tunnels and the position of the bone anchor in the medial aspect of the tibia is important to not cross the tunnels for the CrCrL and CdCrL reconstruction. A transarticular temporary pin is not recommended because of the risk of crossing the bone tunnels.

Based on our results at the 8 week and 6 month follow up, early joint mobilization preserves ROM and results in good joint function. The advantages of early joint mobilization after articular and ligament surgery have been demonstrated in multiple experimental and clinical studies in animals and people.^8^, ^23^, ^24^, ^25^, ^26^, ^27^ However, this well accepted surgical principle has been challenged by a recent study in cats with MLSI where postoperative immobilization after non‐anatomical reconstructions of the ligaments did not affect outcome.^2^ Because of the lack of a control group with joint immobilization, we cannot conclude superiority of our technique. Future studies should aim at comparing techniques requiring immobilization to techniques with anatomical reconstruction and early joint mobility as reported in this study. Preservation of full stifle function maybe particularly important in cats that need significant propulsive forces for jumping.^28^, ^29^

Meniscal repair was performed in 10 cats. In most cases the torn meniscal tissue was not repairable and was resected. The menisci play several essential roles in the stifle joint, including enhancing joint congruity and stability, facilitating load transmission, and providing shock absorption.^30^, ^31^ Under weightbearing, the menisci generate hoop stress, which enables effective load distribution. Conversely, when the menisci are transected or detached, their ability to transmit load is markedly reduced. This loss of function is particularly detrimental in an already destabilized stifle.^30^, ^31^ In human studies, it has been demonstrated that anterior tibial translation increases by approximately 58% in anterior cruciate ligament–deficient stifles when the meniscus is also compromised.^32^ This suggests that meniscal integrity may serve as a prognostic factor in MLSI. However, because nearly all of our cases exhibited meniscal involvement and most required partial or complete meniscectomy, we were unable to assess the specific prognostic impact of meniscal status in this cohort.

In our study the intra‐articular reconstruction of the CdCrL was developed after observing caudal tibial subluxation in three cases without CdCrL treatment. The caudal tibial subluxation may have been exacerbated by early weightbearing. Similarly, the postoperative medial patellar luxation reported in two cats may have been caused by a joint capsule tear due to early postoperative activity. After modifying the technique, the complication rate was reduced from 26% to 8.6% suggesting that the CdCrL reconstruction is effective in improving joint stability even without joint immobilization. Our results compare favorably in terms of complications to what has been reported in previous studies in dogs and cats.^1^, ^2^, ^3^, ^4^ Techniques employing postoperative immobilization may achieve stability without CdCrL reconstruction by developing enough fibrosis to prevent caudal subluxation.

A major limitation of this study was the variability of the injuries and the lack of a control group treated with a non‐anatomical technique with joint immobilization. The variable injury pattern is typical of multiligamentous injuries such as MLSI. We divided the population into three groups, to organize the clinical findings based on the severity of the injury. The lack of a control group is a concern when interpreting the results. However, the study was designed as a pilot to test if coaptationless treatment and internal brace are feasible and safe in cats. Our outcomes lack quantitative evaluation of lameness with gait analysis, but the combination of the surgeon's evaluation and the client assessment help interpreting the results.

## 5 CONCLUSION

In conclusion, our results suggest that a strong multiligament reconstruction using bone tunnels, knotless anchors and braided ultra‐high‐molecular‐weight polyethylene suture offer a safe and effective method for treating MLSI in cats. Postoperative joint immobilization or external coaptation were not used, resulting in good ROM and successful outcome. We recommend an anatomical reconstruction of the CdCrL in case of concurrent CrCrL rupture, to counteract the forces from the reconstruction of the CrCrL.

## AUTHOR CONTRIBUTIONS

Knell SC, DECVS, DVM, PhD: Conception and design, acquisition of data and medical file review, analysis and interpretation of results, drafting the article, revising the article for intellectual content, final approval of the completed article. Schmierer PA, DECVS: Conception and design, acquisition of data and medical file review, analysis and interpretation of data, revising the article for intellectual content, final approval of the completed article. Pozzi A, DVM, MS, DACVS (Small Animal), DECVS, DACVSMR, DECVSMR: Analysis and interpretation of data, revising the article for intellectual content, final approval of the completed article.

## FUNDING INFORMATION

No financial support was received for this study.

## CONFLICT OF INTEREST STATEMENT

The authors declare that all three authors are paid part time consultants for Arthrex GmbH.

## Data Availability

The data that support the findings of this study are available on request from the corresponding author. The data are not publicly available due to privacy or ethical restrictions.

## APPENDIX A

### A1 SURGICAL TECHNIQUE

The surgical technique is based on the principle to reconstruct each ligament (Figure 1):

- CrCL: An extra‐articular reconstruction of the CrCL using a polyfilament suture anchored on bony structures (TR procedure).
- CdCL: An intra‐articular reconstruction of the CdCrL with bone tunnels.
- Medial CL: An anatomical knotless reconstruction.
- Lateral CL: The reconstruction of the CrCL also aided to reconstruct the lateral CL.
- Meniscus: An inside‐out suture technique to preserve joint function.^15^

### A2 EXTRA‐ARTICULAR RECONSTRUCTION OF THE CRCRL AND THE LATERAL COLLATERAL LIGAMENT USING BONE TUNNEL AND MULTIFILAMENT SUTURE

The TR procedure is performed as described by Cook,^13^ adapted to cat anatomy, using a smaller implant than in dogs (MiniTightRope, Arthrex GmbH, Munich, Germany) (Figure 2). To ensure the correct placement of the bone tunnels, the caudal margin of the groove of the long digital extensor and the caudolateral part of the lateral femoral condyle are exposed.^13^

The bone tunnels are drilled with a cannulated 2.0 mm drill bit. This size tunnel does not allow to shuttle the button as described in dogs. The button is removed, and the suture is shuttled with a looped nitinol needle (Arthrex GmbH).

Cats have a shallow groove of the long digital extensor tendon that may be more challenging to palpate than in dogs. Exposure of the long digital extensor is recommended for accurate placement of the tunnel, which should be positioned in the cranial aspect of the extensor groove, approximately 1–2 mm distal to the joint level. The exit of the tibial bone tunnel should be placed distally to the insertion of the medial collateral ligament to avoid interference with the knotless anchor used for the reconstruction of the medial collateral ligament and caudal to the exit tunnel of the CdCrL reconstruction to avoid tunnel crossing.

The shape of the femoral condyle of the cat is oval, with the short axis directed proximo‐distally. This anatomical feature results in a narrow window for the femoral drill tunnel, which requires accurate guide wire placement. Figure 1B demonstrates the correct placement of the tunnel in the center of a triangular area bordered by the fabello‐femoral joint proximally, the articular surface of the femur caudo‐distally, and the lateral CL cranially. Care must be taken to maintain enough distance to the articular surface to avoid damage while over‐drilling the guide wire with the cannulated drill bit. The exit of the femoral bone tunnel should be placed in the center of the medial aspect of the femoral metaphysis to prevent interference with the femoropatellar joint and maintain sufficient bone stock around the tunnel.

After preparing both bone tunnels, a sterile adhesive surgical drape (Ioban 3M Switzerland GmbH, Rueschlikon, Switzerland) is applied to the surgical wound to avoid contact of the implant with the skin, and the surgical gloves are changed before handling the suture. The implant is disassembled, and a nitinol lasso (Arthrex GmbH) is used to place a shuttling suture. After completing all tunnels, the looped suture is used to shuttle both strands of the mini‐TR (Arthrex GmbH) through the tibial and femoral tunnels, securing the knot and the button on the medial side of the distal femur (Figure 2E). The mini‐TR is hand‐tensioned and knotted as described.^13^ Tension is applied until the drawer sign is neutralized without eliciting external tibial rotation as controlled by visual control.

### A3 INTRA‐ARTICULAR RECONSTRUCTION OF THE CDCRL USING BONE TUNNELS AND MULTIFILAMENT SUTURE

The anatomical reconstruction of the CdCrL consists of a braided ultra‐high‐molecular‐weight polyethylene suture fixed through bone tunnels drilled at the ligament footprint similar to what was published in humans and dogs (Figure 3).^35^, ^36^

First, an aiming guide is used to insert a guide wire from the cranio‐medial aspect of the tibia, exiting at the tibial footprint. The tibial tunnel is drilled from outside‐in (cranial to caudal) with a 2.0 mm cannulated drill bit, at a 60° angle relative to the tibial plateau. The femoral tunnel is drilled from the footprint of the CdCrL exiting proximo‐medially to the trochlear groove. After preparing both bone tunnels, a sterile adhesive surgical drape (Ioban 3M Switzerland GmbH) is applied to the surgical wound to avoid contact of the implant with the skin, and the surgical gloves are changed before handling the suture. A temporary looped suture is shuttled using a nitinol needle. A single strand of polyfilament suture is shuttled using the looped suture from the femur, proximo‐distally, to the tibia. The suture is tied over a button on the tibia, with the stifle at 90° flexion angle. Care is taken to avoid interference with the previously drilled bone tunnels for the extraarticular reconstruction of the CrCrL (Figure 1B).

### A4 PROSTHETIC RECONSTRUCTION OF THE MEDIAL CL WITH A KNOTLESS ANCHOR

A 2.0‐mm diameter monocortical hole is drilled in the center of the femoral and tibial footprints of the medial CL with a drill stop set to a depth of 10 mm (Figure 4). Care is taken to angle the femoral drilled hole away from the joint level. The cortex is over‐drilled with a 2.5‐mm drill bit to facilitate the introduction of the knotless anchor (PushLock, Arthrex GmbH) (Figure 4B). Care must be taken not to over‐drill the entire bone tunnel, as this might reduce pull‐out strength.

A strand of #0 Fiberwire (Arthrex GmbH) or a 1.3 mm Suture‐Tape (Arthrex GmbH) is introduced in the eyelet of a 2.4‐mm PushLock (Arthrex GmbH). The PushLock is seated in the femoral hole and, according to the manufacturer's instructions, inserted to the level of the first laser line with the help of a mallet. After removing the protecting cap, the PushLock is inserted completely via the advancement of the cannula until the PushLock is flush with the bone. The procedure is repeated for the tibia with the stifle at a standing angle and in a slight varus position. The polyblend suture is kept under tension while inserting the second PushLock by maintaining constant traction on the suture during insertion. Stability is ensured, and the surgical site was copiously lavaged before closure (Figure 2F).

In very active or heavy cats and when bone stock allowed, a larger anchor (Arthrex GmbH) is used for both femur and tibia to enhance construct stability (bone tunnel 2.8 mm). Because of the limited bone stock for the femoral anchor, the tunnel should be drilled precisely and should not be greater than one third of the condylar width to decrease the risk of iatrogenic condylar fracture. Additionally, the use of a longer anchor necessitates precise placement to avoid encroachment into the femoral notch. In the tibia, the bone tunnel must be drilled bicortically to ensure the anchor does not interfere with the transcortex and can be inserted fully.

### A5 CAPSULAR AND MENISCAL TEARS

Traumatic tears of the joint capsule are repaired using monofilament suture material (3–0 polydiaxanone, Monoplus, B. Braun, Tuttlingen, Germany) with a simple continuous suture pattern (Figure 5). Meniscal injuries are treated with partial, total meniscectomy or meniscal repair (Figure 5) based on the type of tear.^4^, ^33^, ^34^ Peripheral tears are repaired with a horizontal suture using 2–0 PDS. A total meniscectomy is performed if the meniscus tissue is damaged and non‐functional.

### A6 SUTURE TENSIONING

Achieving an anatomical position of the joint during tensioning is crucial to establish long term joint function.

The following order was found to re‐establish joint alignment most reliably with the leg held in standing angle (120°–140°):

- Drilling of bone tunnels for the CdCrL and CrCrL
- Placement of the sutures through the desired bone tunnels
- Treatment of meniscus (resection or reattachment)
- Tightening the CdCrL using a shoestring loop manually without tensioner
- Tightening the CrCrL using a shoestring loop manually without tensioner
- Final knotting of CdCrL and CrCrL
- Insertion and fixation of the knotless anchors for the medial CL.
